# Minimally Invasive Root Canal Cleaning: Evaluating Supplementary Irrigation Techniques

**DOI:** 10.3390/dj13050192

**Published:** 2025-04-27

**Authors:** Alissa Tiscareño, P. S. Ortolani-Seltenerich, Ana Ramírez-Muñoz, Omar Pérez-Ron, Pedro M. Mendez S, Carmen Leal-Moya, Giulia Malvicini, Gaya C. S. Vieira, Alejandro R. Pérez

**Affiliations:** 1 Centro Universitario de los Altos, Guadalajara University, Tepatitlán 47600, Mexico; alissastm@hotmail.com (A.T.); endo@cualtos.udg.mx (C.L.-M.); 2 Department of Dental Pathology and Therapeutics, Faculty of Dentistry, UCAM, 30107 Murcia, Spain; psortolani@gmail.com; 3 Department of Endodontics, Rey Juan Carlos University, 28922 Madrid, Spain; anaramimu96@gmail.com; 4 Member of Research of Endodonto23 Group, Miami, FL 33326, USA; ceosj@hotmail.com; 5 Private Practice in Porto, 4400-239 Porto, Portugal; od.pedromendez@gmail.com (P.M.M.S.); dragayavieira@gmail.com (G.C.S.V.); 6 Unit of Endodontics and Restorative Dentistry, Department of Medical Biotechnologies, University of Siena, 25020 Siena, Italy; giulia.malvicini@student.unisi.it

**Keywords:** chemomechanical preparation, cleaning effectiveness, diode laser, passive ultrasonic irrigation, root canal

## Abstract

**Objectives:** This study aimed to evaluate the efficacy of cleaning in minimally shaped mesial and oval distal canals of 3D models of mandibular molars, focusing on positive pressure irrigation, wireless and conventional passive ultrasonic irrigation (PUI), and diode laser (DL) at 980 nm. **Methods:** Forty-four 3D-printed resin models, based on eleven natural mandibular molars (each with mesial and distal canals), were divided into four groups (*n* = 11 per group) to evaluate different irrigation methods. A total of 22 mesial canals (size 20/.04) and 11 oval distal canals (size 25/.04) were analyzed per group. Each root canal was uniformly filled with an artificial hydrogel to simulate a biofilm mixture. Following this preparation, the specified irrigation techniques were applied to the respective groups. Quantitative evaluations of pre- and post-irrigation images were performed to assess the efficiency of tissue removal along the entire length of the canal and in the apical, middle, and coronal thirds. **Results:** The findings revealed no significant differences in the initial amount of tissue between the samples, indicating uniform filling. In the apical region of mesial canals, conventional PUI showed the highest cleaning efficiency (14.1% residual tissue), significantly outperforming the other methods (*p* < 0.05). Cordless PUI and DL also surpassed positive pressure irrigation, leaving 30.4% and 29.3% residual tissue, respectively, compared to 42.2% with positive pressure. In the middle third, all methods tested performed better than needle irrigation (*p* < 0.05), but there were no significant differences in the coronal third or over the full canal length. Distal oval canals showed no significant differences in cleaning effectiveness among methods. **Conclusions:** Although no single method was superior regarding the full canal length, supplementary techniques such as PUI and DL offer potential benefits over conventional irrigation methods, particularly in the apical third of the canal. Complementary approaches such as conventional PUI and diode laser at 980 nm showed superior cleaning efficiency, particularly in the apical third. These results suggest their integration could improve the effectiveness of cleaning in minimally instrumented mesial canals.

## 1. Introduction

The primary goal of endodontic treatment is to eradicate infections from the root canal system to promote the healing of the periradicular tissue [1]. Central to this process is chemomechanical preparation, which combines mechanical instrumentation and chemical irrigation [2]. This combination is crucial for effectively removing bacterial biofilm, necrotic tissue, and debris from the root canal and is essential for the success of the endodontic procedure [1,2].

However, the complex anatomy of the root canal system often poses a challenge to the effectiveness of the chemomechanical preparation [3]. Features such as canal curvatures, isthmuses, lateral canals, and apical ramifications may result in incomplete preparation by standard instruments, leaving areas of the canal wall untreated [2]. Studies have shown that in some cases, between 18% to 29% of the canal surface can remain unprepared [2,3], thus serving as a reservoir for tissue and bacterial persistence.

A recent study [3] has corroborated these findings by evaluating the morphological conditions of canal surfaces that remain uninstrumented. Using a correlative approach combining micro-computed tomography (micro-CT) and microscopy, the study revealed that apart from the coronal part of canals with vital pulp, the majority of non-instrumented areas in both vital and necrotic teeth contained tissue remnants and/or bacteria.

The survival of bacteria after extensive chemomechanical preparation remains a significant concern [2,4]. Studies have shown that tissue and bacteria remain in regions not reached by the instruments or where irrigants like sodium hypochlorite (NaOCl) are less effective [2,3,5]. Clinical, bacteriologic studies found bacteria in approximately 30–60% of canals after chemomechanical preparation [6,7,8], posing a post-treatment apical periodontitis risk due to incomplete root canal system disinfection [9].

This problem is partly due to the limitations of conventional needle and syringe irrigation techniques, which may only reach some areas within complex anatomical structures such as isthmuses, particularly in mandibular molars [2,3,10]. An isthmus, a narrow connection between two root canals [11], occurs in 17 to 100% of mandibular molars [12,13]. Cleaning and disinfecting the isthmus is challenging [5], and due to physical limitations, it is almost impossible for instruments to reach the isthmus and other remote areas of the canal system. Consequently, the cleaning and disinfection of these areas depends mainly on the chemical effects of irrigants [5].

Another significant challenge that can hinder the effectiveness of endodontic treatment is dealing with oval root canals [10]. The presence of oval canals presents a formidable obstacle when it comes to thoroughly cleaning, shaping, and disinfecting the root canal system, mainly when rotary instruments are used for the preparation process [10,14]. This challenge arises because rotary instruments typically create a round cross-sectional configuration, leaving recesses unprepared at the ends of the largest diameter of the oval canal [10]. Oval canals are common in certain types of teeth, such as mandibular incisors, maxillary second premolars, and the distal root of mandibular molars [15]. Studies using micro-CT scans have shown that an unprepared canal surface in oval canals could vary from 17% to 80%, depending on the instrumentation techniques used [10,16].

To overcome these limitations, there has been an increasing focus on incorporating supplementary techniques to enhance disinfection [5,6]. These include various forms of ultrasonic activation and lasers [5,17], which have been shown to potentially improve irrigation performance and reach complex anatomical areas of the canal.

Passive ultrasonic irrigation (PUI) has emerged as a significant advancement in endodontic irrigation [18]. This approach is recommended to activate and agitate the irrigating solution by generating acoustic streaming and cavitation. The advent of new cordless ultrasonic activation devices has further enhanced this aspect of endodontic therapy, providing clinicians with more user-friendly options. However, the efficacy of additional PUI treatment with NaOCl, as shown in in vitro and in vivo studies, has shown inconclusive results [5,6].

Diode lasers (DLs), used in endodontics for tissue dissolution in root canal treatments, have also attracted considerable attention. DLs, e.g., with a wavelength of 980 nm, have shown promising results in activating root canal irrigants, enabling efficient tissue dissolution in the root canal system [17]. DLs operate by inducing a rapid temperature increase, triggering the irrigant and forming vaporized bubbles, which enhance soft tissue dissolution [17].

Numerous studies have investigated the disinfection capacity of DLs at wavelengths of 655–810 nm in conjunction with a photosensitizer for photodynamic therapy to optimize intracanal disinfection [19,20]. However, the cleaning effectiveness of DLs at a wavelength of 980 nm compared to other methods such as PUI or conventional needle and syringe has not yet been investigated. The 980 nm DL, although less commonly used, exhibits favorable physical properties, including deeper tissue penetration and lower light scattering in the near-infrared spectrum [21,22]. Unlike shorter wavelengths, it acts primarily through photothermal activation, promoting irrigant agitation and potential biofilm disruption [17,23]. Additionally, its application with upconversion nanoparticles has shown the enhanced production of reactive oxygen species [22,24,25]. These attributes support the rationale for investigating the 980 nm DL as a supplementary irrigation method, particularly in minimally shaped or anatomically complex canals.

An important trend in endodontics is the adoption of a minimally invasive treatment approach (MIT). MIT emphasizes the preservation of structural dentin and aims to increase fracture resistance and prolong the longevity of endodontically treated teeth [26]. It is worth noting that removing dentin from the root canal may lead to a redistribution of stress towards the apical region, which in turn may reduce the tooth’s resistance to flexural forces and increase the risk of fracture [26]. Nevertheless, it is essential to recognize that this conservative preparation method is associated with limitations. This is mainly because many conventional irrigation techniques rely on larger preparations for optimal fluid dynamics and antibacterial effects [27,28].

Therefore, there is a crucial need to develop irrigation techniques consistent with the principle of treating minimally prepared root canals. To maintain the mechanical efficiency of the MIT approach, it seems essential to carefully incorporate extended conservative access cavities and minimize the removal of root dentin during shaping while ensuring that the cleanliness of the root canal system remains uncompromised.

The null hypothesis of this study posits that there is no significant difference in cleaning effectiveness among various irrigation techniques, specifically positive pressure irrigation, cordless and conventional passive ultrasonic irrigation, and diode laser at 980 nm, in minimally prepared root canals.

This study evaluated the cleaning effectiveness of positive pressure irrigation (control), wireless and conventional PUI, and DL at a wavelength of 980 nm in minimally prepared root canals using mesial and oval distal canals of 3D resin replicas of mandibular molars of natural teeth.

## 2. Materials and Methods

### 2.1. Sample Preparation

The sample size was determined using G*Power 3.1 software (Heinrich Heine Universität, Düsseldorf, Germany) based on the results of a previous study [29]. Assuming a significance level of 0.05, a statistical power of 80%, and an effect size of 0.4 (Cohen’s f), the analysis indicated that at least 9 3D dental replicas per experimental group were necessary. The present study received ethical approval from the local ethics committee of Universidad Rey Juan Carlos, Madrid, Spain (N. 1702202308023).

Eleven 3D resin replicas of natural mandibular molars were obtained (Surpreendente 3D tooth, Vila Nova de Gaia, Porto, Portugal). These teeth were selected according to specific criteria: Mesial Vertucci Class II mandibular molars with moderately curved roots (<20°), consistent with an isthmus between both roots in the apical region and oval distal canals, apical diameters not exceeding size #15 for mesial canals and #25 for distal canals, and lengths between 20 and 21 mm. For a canal to be classified as oval-shaped, the buccolingual diameter was required to be at least twice the mesiodistal diameter.

The mesial and distal canal replicas were instrumented using One RECI 20/.04 (Coltene Whaledent, Altstätten, Switzerland) for the former and 25/.04 for the latter at 300–400 rpm, 1.2 N and 170°/60°, according to the manufacturer’s recommendations. They were then rinsed with 2 mL of distilled water during instrumentation using a 30 gauge (G) Navitip needle (Ultradent, South Jordan, UT, USA) that was positioned 3 mm from the working length (WL).

After instrumentation, the replicas were scanned with micro-CT. This resulted in eleven different mandibular molar anatomies, enlarged to 20/.04 in the mesial canals and 25/.04 in the distal canals, which were subsequently printed. This procedure eliminated biases associated with the instrumentation for each anatomy.

### 2.2. Micro-CT Scanning

The 3D replicas were scanned after chemomechanical preparation using a Phoenix Vitomex S240 micro-CT scanner (General Electric, Boston, MA, USA). The scan parameters included an isotropic resolution of 20.0 mm, 105 kV, 70 mA, a full 360-degree rotation around the vertical axis, and a 0.1 mm thick filter, resulting in a scan time of approximately 25 min per replica. These scans were reconstructed using Phoenix-x V |tome|x S240 3D software (General Electric, Boston, MA, USA). A total of 1250 images per replica were generated with settings such as ring artifact correction at 8, beam hardening correction at 50%, and smoothing set at 6. STL files were generated from these reconstructions using 3D Slicer 5.0.3 software (http://www.slicer.org) to support the internal visualization of the individual anatomical structures.

### 2.3. Manufacture of 3D Replicas in Resin

The STL files of the 3D replicas were imported into AnyCubic PhotonWorkshop software (https://www.anycubic.es) to determine the exact positioning and support required for the printing process. Eleven anatomically identical replicas were then placed in the reservoir of the Anycubic Photon Mono M5s printer (Anycubic Technology Co., Shenzhen, China), which has a resolution of up to 10 μm. The printing file, stored on a SanDisk USB flash drive (Milpitas, CA, USA), was used in the printer, which was filled with 200 mL of transparent water-soluble resin (Anycubic Technology Co., Shenzhen, China) with a wavelength of 365–405 nanometers. The printing process took about 90 min. The distal root of each 3D replica was separated to avoid root overlap and noise in the final image analysis.

After printing, the replicas were cleaned in hot water using the Anycubic Wash & Cure Plus device (Anycubic Technology Co., Shenzhen, China) to remove excess resin and subjected to a 20 min polymerization process for final curing. For a comprehensive comparative analysis, each group included 11 3D resin replicas, each with two mesial canals (*n* = 22) and one distal canal (*n* = 11).

### 2.4. Tissue Simulation with Hydrogel Model

A 30G NaviTip needle (Ultradent Products, Inc., South Jordan, UT, USA) was used to evenly fill the canal system with an artificial biofilm mixture (AB) formulated with a hydrogel. The hydrogel, which was in a liquid state at the time of injection, had excellent capillary properties and provided a uniform distribution with no voids throughout all canal areas.

The hydrogel was prepared as described by Robinson, et al. [30], dissolving 3 g of gelatin (Merck, Darmstadt, Germany) and 0.06 g of hyaluronan (sodium hyaluronate 95%, Fisher, Waltham, MA, USA) in 45 mL of deionized water at 50 °C. In addition, 0.25 g of red food dye (Condi Alimentar, Camarate, Portugal) and 0.1 g of hollow glass beads (Sigma Aldrich, Bornem, Belgium) were added to improve the visibility of AB. The hydrogel was kept at 30 °C before injection and solidified within 1 min at room temperature, a process that was checked for each sample to ensure consistency.

### 2.5. Experimental Groups

**Figure 1:**
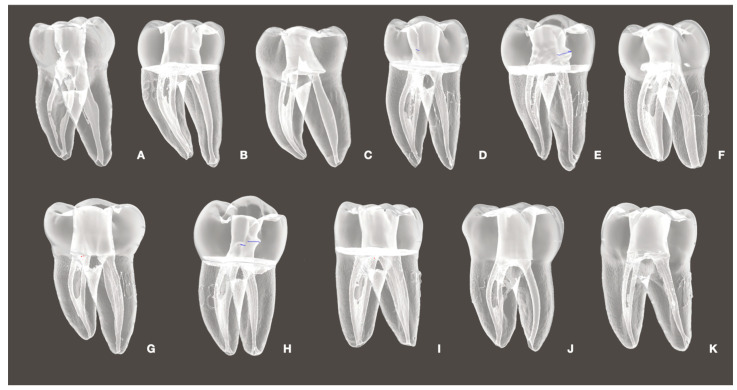
The eleven anatomically distinct mandibular molars selected for this study. Each tooth (**A**–**K**) was scanned using micro-CT and replicated four times via 3D printing, resulting in a total of 44 standardized resin models. These anatomies were used to preserve clinical variability while ensuring controlled experimental conditions across irrigation groups.

**Figure 2:**
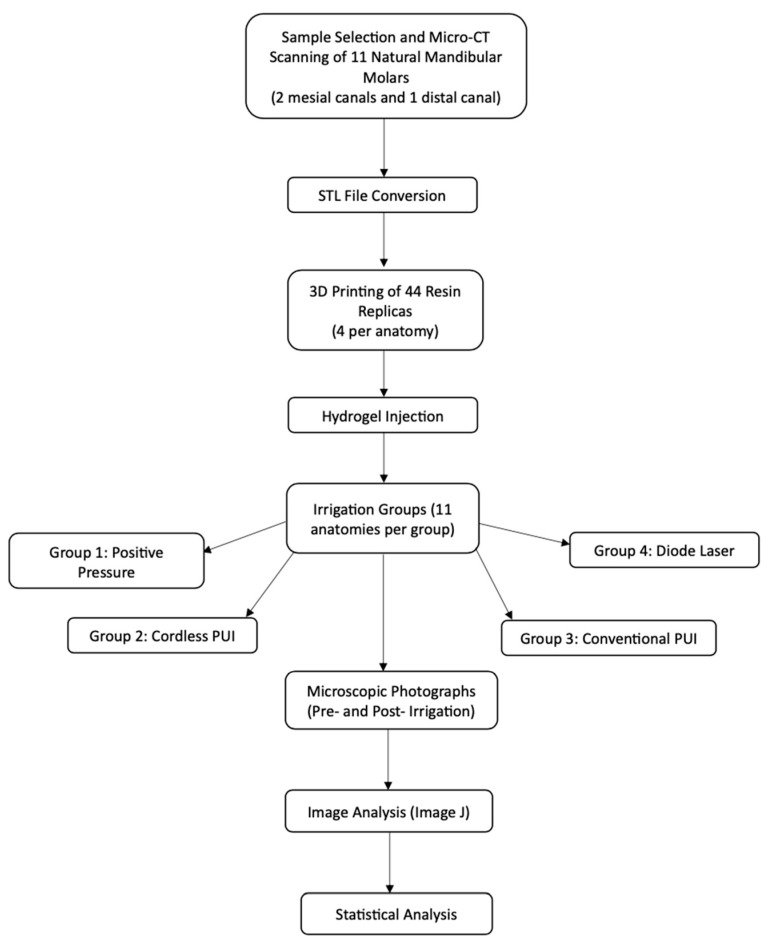
Schematic representation of the experimental workflow.

Forty-four 3D-printed mandibular molar models, representing eleven distinct anatomies replicated four times (Figure 1), were divided into four experimental groups (*n* = 11). Each group received a different irrigation protocol applied to mesial (*n* = 22) and oval distal (*n* = 11) canals (Figure 2):

1. Positive Pressure (Control) irrigation with an open-ended 30G needle positioned 3 mm up from the WL.
2. Cordless PUI Ultra X (Eighteeth, Changzhou, Jiangsu, China), with two activation cycles of 30 seg using a silver tip size of 20/.02 positioned 2 mm up from the WL.
3. Conventional PUI (Varios 370 Lux, NSK America Corp., Hoffman Estates, IL, USA) with two activation cycles of 30 seg using a Irrisafe 20/.02 tip (Acteon, Merignac, France) positioned 2 mm up from the WL.
4. DL at 980 nm with a power of LX16 plus (Woodpecker Medical Instrument Co., LTD, Guangdong, China) and a 0.2 mm tip positioned 3 mm up from the WL. Two activation cycles of 30 s were performed during the procedure.

After initial photographic documentation, the apical foramen was sealed with TopDam (FGM, Joinville, SC, Brazil) to simulate the vapor lock effect. The teeth were then mounted vertically on a holder designed for intracanal procedures. Each canal was rinsed with 2 mL of water in the mesial roots, followed by supplementary procedural steps. This process was repeated in each canal to reapply the adjunctive methods to improve tissue debridement. For the group using needle and syringe, irrigation was performed with 4 mL of water per canal, using continuous in-and-out movements with an amplitude of 3–4 mm. A total volume of 4 mL of water was used in each canal, but the apical third ultimately received 8 mL due to confluent anatomy.

In contrast, in the distal canals, an initial volume of 4 mL of water was introduced, followed by the initiation of the first 30 s cycle. A further 4 mL was introduced into the canal to activate and agitate the solution. Continuous irrigation was performed in the group using the traditional needle and syringe technique, and 8 mL of irrigation fluid was administered. A single operator, specialized in endodontics, conducted the entire procedure.

### 2.6. Tissue Cleaning Efficiency

Before and after irrigation, each model underwent microscopic photography (Carl Zeiss, Berlin, Germany) using a special holder to ensure uniform positioning. The initial and final photographic images were opened in the Keynote program (Apple Inc., Cupertino, CA, USA), and the same size and position were confirmed by superimposing the images. The anatomical structures, such as dentin, were eliminated from the images, leaving only the red-colored root canal system (hydrogel tissue model) in the initial and final samples. For the evaluation, consideration was given to the fact that both main canals had a single apical foramen and an isthmus zone in the apical region connecting both root canals.

These modified images were integrated into ImageJ 2.16.0/1.54p software (National Institutes of Health, Bethesda, MD, USA) to create binary images, facilitating the quantification of the tissue surface area (mm^2^) across the entire canal and in the apical, middle, and coronal thirds. The percentage of remaining tissue for each needle group was calculated based on the differences between the pre-and post-irrigation images.

### 2.7. Statistical Analysis

The data distribution was assessed using the Shapiro–Wilk test and graphical methods. Since the data were normally distributed, one-way ANOVA was used to compare the quantitative assessment of the remaining tissue both between groups (intergroup comparisons) and within each group (intragroup comparisons among the apical, middle, and coronal thirds). Levene’s test was applied to confirm the homogeneity of variances. When significant differences were found, Bonferroni post hoc tests were performed. All statistical analyses were carried out using SPSS software (version 21.0; IBM Corp., Armonk, NY, USA), with the significance level set at 5%.

## 3. Results

Table 1 presents the data from the quantitative analysis of the remaining tissue, showing no significant differences (*p* > 0.05) in the initial amount of tissue between the 3D replicas, suggesting a uniform and comparable filling of the samples.

**Figure 3:**
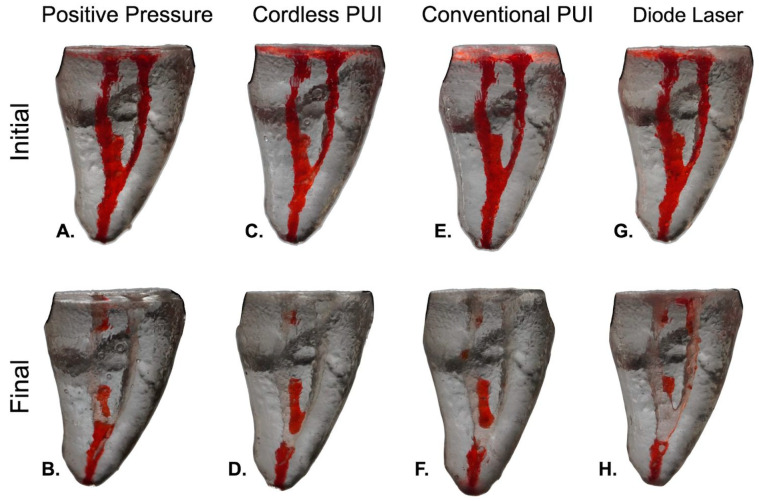
Representative images of mesial root canals instrumented to size 20/.04 and with the presence of a hydrogel model before (**A**,**C**,**E**,**G**) and remaining tissue in the apical third (expressed in percentage) after (**B**) using Positive Pressure (42.2%), (**D**) Cordless PUI (30.4%), (**F**) Conventional PUI (14.1%), and (**H**) Diode Laser (29.3%).

In the mesial canals (Figure 3), no significant differences in cleaning efficiency were found between the four groups in the full canal length (*p* > 0.05). About the apical region of the canal, conventional PUI showed a significantly better cleaning performance in the mesial canals of mandibular molars compared to the other groups (*p* < 0.05). Specifically, the percentages of residual tissue were as follows: 42.2% for positive pressure irrigation with needle and syringe, 29.3% for DL, 30.4% for cordless PUI, and 14.1% for conventional PUI, as shown in Figure 2 and detailed in Table 1.

Furthermore, wireless PUI and DL exhibited an enhanced cleaning efficiency compared to conventional needle irrigation (*p* < 0.05), with no significant differences in cleaning efficiency observed between DL and wireless PUI (*p* > 0.05).

In the evaluation of the middle third, the analysis revealed no significant differences between the activation techniques (*p* > 0.05). Nevertheless, all methods proved significantly more effective than conventional irrigation with needle and syringe (*p* < 0.05). In the coronal third, the study revealed no significant differences in the cleaning ability of the root canals between the groups (*p* > 0.05).

**Figure 4:**
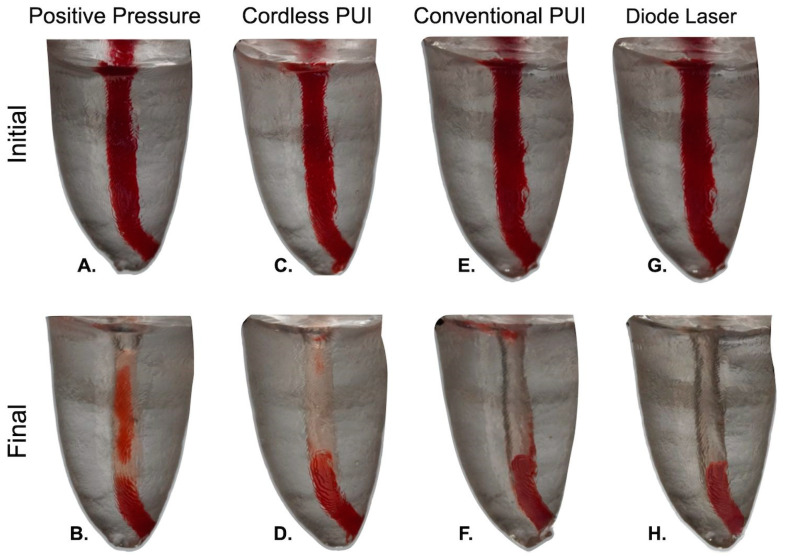
Illustrative visuals of remaining tissue in the apical region (expressed in percentage) with the different adjunctive approaches in oval distal canals. Images were taken before (**A**,**C**,**E**,**G**) and after irrigation with (**B**) Positive Pressure (42.3%), (**D**) Cordless PUI (41.2%), (**F**) Conventional PUI (40.3%), and (**H**) Diode Laser (35.2%).

When analyzing the distal oval canals (Figure 4), the percentage of remaining tissue over the entire length of the canal was found to range from 37.4% to 47.2% for the needle irrigation and DL. Comparable percentages were observed for the conventional PUI and wireless PUI groups at 38.4% and 39.4%, respectively (see Table 2). In contrast, the irrigation efficiency in the apical region varied between 41.2% in the cordless PUI and 40.3% in the conventional PUI group. Meanwhile, the percentages for positive pressure irrigation and the DL method were reported as 42.3% and 35.2%, respectively. The statistical analysis revealed no significant differences between the groups regarding the cleaning effectiveness, neither across the full canal length nor in any third of the canal (*p* > 0.05).

## 4. Discussion

The objective of this study was to evaluate the cleaning effectiveness of different agitation and activation techniques in root canals that have undergone conservative preparation.

The analysis has primarily highlighted the limitations of using traditional needles and syringes for root canal irrigation. Although these conventional methods are commonly used, the present study shows their suboptimal cleaning efficiency when minimal instrumentation was performed in complex anatomies, especially in the apical third of the root canal.

Conventional root canal irrigation with positive pressure is mainly limited by its inability to remove tissue thoroughly [3], particularly in complex root canal anatomies [2]. This study emphasizes this limitation, showing that conventional needle irrigation is significantly less efficient at tissue removal than alternative techniques, leading to the rejection of the null hypothesis. This discrepancy is especially evident in the apical region of the mesial canals of mandibular molars, where conventional needle irrigation leaves a significantly higher percentage of residual tissue (42.2%) compared to adjunctive approaches.

The findings suggest that conventional needle irrigation remains a foundational technique during chemomechanical preparation. Still, it is essential to emphasize that it needs to be complemented by more advanced techniques such as PUI or DL, specifically when dealing with teeth featuring intricate anatomical features and minimal instrumentation [31].

However, it is important to point out that in the present study, the mesial canals were instrumented up to a size 20/.04. The instrumentation of the root canal to a larger size, such as #35, is crucial for several reasons when using a needle and syringe for irrigation [27,32]. Firstly, enlarging the canal size facilitates greater fluid dynamics, allowing for increased flow and the better access of the irrigant to the canal’s entirety [27], including its apical region [32]. This is particularly important because the effectiveness of needle and syringe irrigation heavily relies on the physical flow of the irrigant to remove tissue debris and disinfect the canal [33]. Larger instrumentation sizes create a wider canal space, improving the penetrability and flow of the irrigant [28], thus enhancing the renewal and exchange of the solution within the canal system [34].

The mechanical limitation of a needle and syringe is primarily its inability in narrower canals to agitate the irrigant sufficiently within the complex root canal system and achieve optimal flow [35], especially in the apical third, reducing its efficacy in tissue removal and disinfection [28].

The study’s results indicate that conventional PUI outperformed other methods in the mesial canals of mandibular molars, with significantly less residual tissue (14.1% for conventional PUI compared to 42.2% for needle and syringe, 29.3% for DL, and 30.4% for cordless PUI), highlighting the effectiveness of PUI in challenging areas. The results of this study agree with another where greater cleaning was observed when ultrasonic activation was used in canals with smaller apical preparations compared to using only a conventional syringe [31].

Surprisingly, traditional PUI performed significantly better than wireless PUI in the apical region of the root canal. Although both methods are based on similar principles, the results of this study suggest that conventional PUI has greater activation and agitation capabilities than wireless PUI. The superior performance of traditional PUI over cordless systems observed in the apical region can be explained by differences in energy delivery and fluid dynamics.

Conventional PUI devices are connected to a mains-powered ultrasonic unit that provides stable high-frequency, high-amplitude vibrations. This efficient transmission of energy leads to more intense acoustic streaming and cavitation, both of which are essential for dislodging debris and the smear layer from narrow apical canal spaces [18,36]. The consistent oscillation also supports the formation and collapse of microbubbles, critical for penetrating complex anatomies [37]. In contrast, cordless systems often rely on battery power and miniaturized components, which may result in lower vibrational amplitude and reduced energy transmission. This can diminish the cavitation efficiency and reduce irrigant movement, ultimately limiting the effectiveness of cleaning in the apical third [36]. These mechanical limitations likely explain the significantly higher residual tissue observed in the cordless PUI group. As such, the choice of activation device may play a critical role in optimizing irrigation outcomes, especially when dealing with minimally instrumented or highly curved canals. Future studies should validate these findings, evaluating the wavelengths and powers of both methods.

The similar performance of wireless PUI and DL, both showing enhanced efficiency over needle irrigation, underscores the importance of selecting advanced irrigation methods to optimize root canal cleaning. PUI, by generating acoustic streaming and cavitation effects [18], and DL, by activating irrigants and inducing tissue dissolution through rapid temperature increases and the formation of vaporized bubbles, [17] may improve the cleaning efficacy. These advanced techniques facilitate the deeper penetration of irrigants into difficult-to-reach areas, significantly improving the effectiveness of cleaning in the root canal system [17,31].

In the middle third of the root canals, all activation techniques proved significantly more effective than conventional irrigation with a needle and syringe, underlining the latter’s inefficiency in removing tissue, probably because of the narrower space that limits their action. However, no significant differences were found among the activation methods in this region. This indicates that while activation methods are advantageous, the choice between them may not significantly impact cleaning outcomes in the middle third of the root canals.

In the coronal third, the study did not reveal significant differences in cleaning ability among the various groups. This suggests that conventional needle irrigation may be comparably effective in this region to activation methods, and the choice of technique may be less critical.

However, caution is required when interpreting the current study’s findings. First, no comparison was made between smaller and larger apical diameters to determine whether activation and agitation techniques could replace apical enlargement in the mesial roots of mandibular molars or whether they should merely serve as supplementary methods to improve tissue removal and disinfection efficacy.

Second, the main goal in managing apical periodontitis is the eradication of bacterial biofilm in the root canal system [1]. However, increasing emphasis is being placed on minimally invasive endodontic treatments, which, while increasing fracture resistance [38], could potentially compromise disinfection [39] and, consequently, treatment outcomes.

A previous study showed that when root canals are prepared to an apical size of 20 or 25, NaOCl cannot effectively reach the working length when using syringes and needles [35]. To address this problem, in vitro studies have developed activation and agitation irrigation strategies to ensure the thorough disinfection of minimally prepared canals [31,40]. To date, no clinical studies provide evidence of conducting minimal root canal preparations and relying on agitation and activation techniques to compensate for disinfection.

Existing evidence suggests that larger apical diameters allow better renewal of the irrigant in the apical third of the root canal [28], resulting in enhanced disinfection [41] and, consequently, a higher success rate in endodontic treatment [42]. Future clinical investigations must validate the efficacy of minimally shaped canals in complex anatomies such as mandibular molars using adjunctive approaches, particularly regarding their disinfection ability compared to larger apical diameters. These findings could serve as a surrogate endpoint for the overall success of endodontic treatment.

In this study, an important aspect was comparing eleven different anatomical variations in natural mandibular molar teeth. These molars were divided into four groups, resulting in forty-four 3D resin replicas. This meticulous approach helped us minimize any potential biases arising from anatomical differences, and it is more representative of studies on extracted natural teeth where different anatomies are utilized, as opposed to using a single anatomy repeatedly to compare different methods.

Furthermore, an interesting finding was the consistency in the amount of initial tissue in the samples. Our preliminary analysis revealed no significant differences in the presence of tissue when comparing the different groups. It is also worth noting that the mandibular molars we selected with Class II Vertucci anatomy in the mesial canals and oval-shaped in the distal canals provided a representative challenge for evaluating agitation and activation techniques.

Oval canals present unique challenges due to their shape, which often leaves recesses not adequately reached by rotary instruments [10], resulting in incomplete debridement and inadequate disinfection [43]. This is compounded by the difficulty of achieving thorough irrigation [10], which is crucial for removing tissue remnants and bacterial biofilms from these complex anatomical structures [10,31].

The results of the present study showed that in the distal oval canals, the percentage of tissue remaining along the entire length of the canal ranged from 37.4% to 47.2% for the needle irrigation and DL groups. Comparable percentages were observed for the conventional PUI and wireless PUI groups at 38.4% and 39.4%, respectively, and no significant differences were observed in any third of the canal between the groups evaluated. These findings suggest that none of the techniques studied achieved greater tissue cleaning in oval canals than conventional syringes.

The results of this study highlight the inherent limitations resulting from the canal’s oval shape, which can hinder the cleaning effectiveness of even the most sophisticated irrigation methods. These findings are consistent with previous studies that found no significant differences when using different activation and agitation techniques in oval canals [14,44].

However, this study focused exclusively on mandibular molars, which are known for their complex mesial canal systems and large, oval distal canals. While these anatomies present a relevant and challenging model for evaluating irrigation performance, they do not reflect the full range of anatomical variability seen across different tooth types. For example, anterior teeth and premolars may have simpler or narrower canal systems, which could influence irrigant dynamics and the effectiveness of activation methods. Therefore, caution should be exercised when extrapolating these results to other tooth anatomies. Future research involving a wider array of tooth types is needed to assess whether these findings hold true in different clinical scenarios.

These results are critical for several reasons. First, they suggest that while supplementary techniques can play a role in endodontic treatment, their ability to significantly outperform traditional irrigation methods in oval canals is limited [14]. This limitation is particularly pronounced given the shape of these canals, which inherently complicates the task of comprehensive tissue removal [10]. Secondly, the percentages of remaining tissue suggest that a substantial amount of the canal surface remains uncleaned, regardless of the irrigation technique used [14]. This scenario could compromise the disinfection process, leaving the canal system susceptible to persistent infection and endodontic treatment failure [45].

It is crucial to emphasize that regardless of the specific technique employed or the type of anatomical variation considered, none of the procedures completely removed the tissue, particularly in the apical third of the canal. The apical root canal area is crucial in infection control, as bacteria in this region can contribute to post-treatment apical periodontitis [46]. However, complete tissue removal from this area remains challenging with current techniques. Our findings are consistent with previous studies that have also reported a higher incidence of residual tissue and/or bacteria in the apical region of mesial and distal canals of mandibular molars [2,3,10].

In addition, tissue persistence may interfere with the proper filling of the root canal, which may lead to treatment failure in necrotic teeth [47]. As demonstrated in this study, the main reason for tissue persistence is the limitation of existing techniques in the reliable delivery of irrigants to different regions of the root canal, particularly those with complex anatomical features. These findings emphasize the urgent need for innovative methods that can predictably optimize tissue removal throughout the entire root canal system.

In this study, 3D-printed resin models were used instead of extracted natural teeth. This was a deliberate methodological choice to ensure anatomical standardization and reduce the variability between samples. The models were derived from high-resolution micro-CT scans of eleven distinct mandibular molars to maintain consistency across experimental groups [48,49]. It is acknowledged that 3D-printed replicas cannot fully reproduce the biological properties of dentin, such as the tubule structure or tissue interaction with irrigants. However, they offer several advantages for comparative studies, most notably the ability to eliminate confounding factors like canal size variation, mineralization differences, and previous treatments, which are common in extracted teeth [50]. This makes them a suitable model for evaluating the mechanical cleaning potential of irrigation protocols in a controlled setting. Recent studies have shown that findings from 3D-printed teeth closely simulate those obtained from natural teeth regarding the shaping, obturation, and irrigation performance [29,51,52,53]. While they are not a substitute for clinical trials, these models are increasingly accepted in endodontic research and education as valuable tools for preliminary testing.

This study utilized a previously validated hydrogel-based substrate to simulate intracanal soft tissue [29,30,54]. Hydrogels provide a standardized, reproducible medium that closely mimics the physical properties of soft tissue, allowing for controlled experimental conditions and facilitating the quantitative assessment of cleaning efficacy. Their transparency and compatibility with imaging software further enhance their utility in evaluating tissue removal and irrigant performance under repeatable conditions [54]. However, hydrogels do not replicate the biological complexity of pulp tissue, including variability in tissue density, vascularization, and the presence of mature bacterial biofilms, factors that significantly influence clinical disinfection outcomes [55]. As such, while hydrogels are highly valuable for isolating the mechanical effects of irrigation techniques, caution is warranted when extrapolating these results to in vivo endodontic treatments. Future studies may benefit from combining hydrogel models with biologically active substrates or ex vivo tissue to bridge the gap between experimental control and the clinical setting [55].

Although this study utilized an innovative approach by 3D printing various natural tooth anatomies, it is essential to recognize its limitations. First, water served as an irrigant throughout the experiment, meaning that the evaluation primarily focused on the mechanical aspects of the techniques and did not consider the chemical properties or capabilities of the irrigant. Furthermore, using resin replicas instead of natural teeth may result in variations in irrigant diffusion and tissue removal. In the resin model, there is no buffering activity from the dentin, which occurs in natural teeth when sodium hypochlorite is used. Additionally, the study’s results using distilled water may not fully reflect the clinical reality, potentially underestimating the actual cleaning effectiveness of the tested techniques due to the lack of chemical activity from irrigants like sodium hypochlorite. Finally, the study’s relatively small sample size (*n* = 11 per group), determined through a priori power analysis, was sufficient for detecting significant differences in the apical third of mesial canals (primary outcome), but it may have limited our ability to observe subtle effects in other regions. Larger sample sizes could enhance the statistical power and reduce the risk of type II errors. Moreover, as this is an in vitro study, caution should be exercised when extrapolating these findings to real clinical scenarios.

These findings underscore the importance of adapting irrigation strategies to the anatomical complexity of the root canal system. In clinical practice, areas with limited access, such as minimally shaped or highly curved apical regions, may benefit from the use of advanced activation techniques to enhance irrigant efficacy. Conversely, in more accessible regions of the canal, where mechanical instrumentation and irrigant flow are less restricted, the choice of irrigation method can be more flexible and guided by factors such as the practitioner’s preference, device availability, and cost.

## 5. Conclusions

In conclusion, conventional PUI performed better than other methods in cleaning the mesial canals of mandibular molars, especially in the apical region. Cordless PUI and DL proved to be promising alternatives that offered better cleaning compared to conventional needle irrigation. All methods proved to be superior to conventional irrigation in the middle third of the canal. However, no significant differences were found between the tested groups in the distal oval canals, suggesting that the choice of irrigation technique may have less impact on cleaning efficiency in these canals.

## Figures and Tables

**Table 1:** Residual tissue in mesial canals along the full canal length and the apical, middle, and coronal thirds; expressed as mean (median; range).

Features	Conventional Syringe	Cordless PUI	Conventional PUI	Laser 1W
**Tissue remnants**				
**Full canal**				
Surface (mm^3^)				
Initial surface	17,453.2 (16,862.4; 6997–26,698)	17,168.4 (15,986.3; 5909–28,076)	17,568.1 (16,536.3; 6408–27,458)	17,877.4 (16,398.2; 6205–27,809)
Final	6800.2 (6375.2; 2012–16,034)	5837.4 (5640.3; 0–13,482)	6944.2 (6530.3; 0–14,488)	6625.4 (6909.1; 0–16,164)
Δ%	36.1 (36.3; 11–62)	29.1 (23.2; 0–65)	37.3 (37.2; 0–55)	35.4 (37.4; 0–80)
**Apical**				
**Surface (mm^3^)**				
Initial	3749.3 (3692.4; 933–9456)	3567.3 (3479.3; 628–8413)	3460.2 (3707.4; 107–4763)	3549.1 (3208.2; 853–8514)
Final	1574.3 (870.2; 69–8192)	1246.1 (889.4; 0–3923)	484.6 (398.2; 0–6052)	1035.3 (965.1; 0–4128)
Δ%	42.2 (40.3; 7–87) ^a^	30.4 (25.2; 0–84) ^b^	14.1 (1.3; 0–87) ^c^	29.3 (22.2; 0–85) ^b^
**Middle**				
**Surface (mm^3^)**				
Initial	5708.8 (5679.1; 2301–9934)	5494.2 (5463.4; 2460–9527)	5413.4 (5254.3; 2614–9644)	5277.2 (5086.1; 2795–9547)
Final	1951.3 (1566.2; 0–5603)	745.2 (500.4; 0–4726)	777.2 (694.3; 0–4875)	659.2 (654.3; 0–4769)
Δ%	34.3 (31.1; 0–85) ^a^	13.4 (10.2; 0–53) ^b^	14.3 (12.2; 0–67) ^b^	12.1 (10.1; 0–57) ^b^
**Coronal**				
**Surface(mm^3^)**				
Inicial	8387.7 (8229.2; 3936–11,567)	8470.4 (8342.1; 3857–11,760)	8153.1 (8174.1; 3466–11,834)	8595.4 (8063.2; 3305–11,618)
Final	2995.8 (2557.1; 0–8219)	2559.4 (2483.1; 0–7609)	2453.6 (2396.3; 0–8539)	2590.3 (2457.2; 0–7997)
Δ%	35.1 (34.1; 0–84)	30.4 (29.1; 0–75)	30.2 (33.2; 0–81)	30.1 (34.2; 0–83) ^a^

Note: Different superscript letters within the same row indicate statistically significant differences (*p* < 0.05).

**Table 2:** Residual tissue in distal oval canals along the full canal length and the apical, middle, and coronal thirds; expressed as mean (median; range).

Features (Mean-Median-Range)	Conventional Syringe	Cordless PUI	Conventional PUI	Laser 1W
**Tissue remnants**				
**All canal**				
Surface (mm^2^)				
Initial surface	14,166.2 (13,983.3; 6695–22,107)	14,037.2 (13,019.2; 6416–22,449)	14,587.3 (13,761.2; 6353–22,642)	14,163.3 (13,045.1; 6522–22,445)
Final	6670.1 (4787.1; 606–17,830)	5486.3 (4719.2; 0–13,341)	5570.3 (4676.1; 0–18,473)	5285.2 (4841.2; 0–15,702)
Δ%	47.2 (41.2; 9–88)	39.4 (32.3; 0–85)	38.4 (26.3; 0–77)	37.4 (23.1; 0–91)
**Apical**				
**Surface (mm^2^)**				
Initial	2788.3 (2483.2; 695–5483)	2356.4 (2129.3; 678–5266)	2425.2 (2262.2; 674–5160)	2420.3 (2396.1; 658–5381)
Final	1080.4 (945.3; 216–3131)	951.3 (835.4; 0–3375)	970.1 (801.3; 0–3732)	859.2 (730.2; 0–2549)
Δ%	42.3 (41.2; 13–71)	41.2 (31.3; 0–86)	40.3 (37.2; 0–85)	35.2 (31.2; 0–83)
**Middle**				
**Surface(mm^2^)**				
Initial	5620.3 (4720.1; 1700–8417)	5397.2 (4587.5 (1807–8646)	5558.4 (4565.2; 1622–8599)	5424.1 (4583.4; 1738–8506)
Final	1345.4 (1293.4; 0–5248)	1771.2 (1760.1; 0–5302)	1389.3 (103.2; 0–1168)	1387.4 (1223.1; 0–5919)
Δ%	24.1 (21.2; 0–85)	32.3 (21.2; 0–86)	24.4 (22.1; 0–89)	23.3 (22.2; 0–88)
**Coronal**				
**Surface(mm^2^)**				
Inicial	7636.3 (7098.1; 4795–9894)	7480.2 (7297.3; 4550–9515)	7780.3 (7584.1; 4798–9400)	7713.3 (7576.3; 4355–9502)
Final	3715.2 (3084.3; 0–9255)	3170.2 (2885.4; 0–8616)	3568.4 (3308.2; 0–8692)	3631.2 (3389.1; 0–8415)
Δ%	49.1 (45.3; 0–94)	41.3 (38.2; 0–97)	45.3 (41.2; 0–96)	47.3 (43.2; 0–96)

## Data Availability

The original contributions presented in this study are included in the article. Further inquiries can be directed to the corresponding author.
